# A green metal-free fused-ring initiating substance

**DOI:** 10.1038/s41467-019-09347-y

**Published:** 2019-03-22

**Authors:** Mucong Deng, Yongan Feng, Wenquan Zhang, Xiujuan Qi, Qinghua Zhang

**Affiliations:** 1 Institute of Chemical Materials, China Academy of Engineering Physics, Mianyang, 621000 China; 2 Southwest University of Science and Technology, Mianyang, 621010 China

## Abstract

Over the past century, the search for lead-free, environmentally friendly initiating substances has been a highly challenging task in the field of energetic materials. Here, an organic primary explosive featuring a fused-ring structure, 6-nitro-7-azido-pyrazol[3,4-d][1,2,3]triazine-2-oxide, was designed and synthesized through a facile two-step reaction from commercially available reagents. This organic initiating substance meets nearly all of the stringent criteria of environmentally friendly primary explosives for commercial applications: it is free of toxic metals and perchlorate, has a high density, high priming ability, unusual sensitivities towards non-explosive stimuli, excellent environmental resistance, decent thermal stability, high detonation performance, satisfactory flowability and pressure durability, and is low-cost and easy to scale-up. These combined properties and performance measures surpass the current and widely used organic primary explosive, DDNP. The fused-ring organic primary explosive reported herein may find real-world application as an initiating explosive device in the near future.

## Abstract (web summary)

The search for environmentally friendly, lead-free primary explosives continues to be important for both military and civil applications. Here the authors synthesize an organic fused-ring initiating substance that possesses many of the attributes necessary for commercial application.

## Introduction

Primary explosives, or initiators, represent a class of sensitive energetic materials that can be easily detonated by a small external stimulus such as flame, heat, impact, friction, electric spark, etc. They are widely used in industrial initiating explosive devices (IEDs) like primers and detonators for both military and civil applications such as for use in rockets, satellites, weapons, mining, and tunneling. The first substance historically used as a primary explosive was mercury fulminate (MF) which was discovered in 1628^1^. In the early 1900s, lead azide (LA) and lead styphnate (LS) were identified as good substitutes for MF in military applications, starting the era of lead-based primary explosives^2^. Even now, LA and LS are still the most widely used primary explosives. However, growing concerns over lead contamination stimulate the search for new lead-free primary explosives^3^.

Looking back upon the historical development of lead-free primary explosives (see Table 1), both inorganic energetic metal complexes and organic explosive substances have been explored^4–17^. For most metal-based primary explosives, either perchlorate (ClO_4_^−^) or heavy metals (Co, Ni, Cu, Zn, Cd, Hg, etc.) are inevitably contained, which have been listed as priority pollutants by the US and the European Union^18,19^. Moreover, the potassium-based primary explosives, despite being less toxic, are rarely used due to their complex synthesis or poor priming ability^20–23^. In the quest for lead-free primary explosives, the most effective path forward is the development of organic alternatives, which can completely eliminate the environmental hazards caused by heavy metals while maintaining high priming performances^24^. For example, several latest highly sensitive compounds possess high densities and excellent detonation properties, showing some extent potential as primary explosive^24–29^. Especially a metal-free polyazido compound reported by Chen et al. exhibits excellent initiating efficiency^24^. Unfortunately, most organic substitutes developed for use as primary explosives are hindered for use in large-scale application due to their high sensitivity, cumbersome synthesis, or poor stability^24–29^. To our knowledge, the only widely used organic primary explosive in commercial IEDs is 2-diazo-4,6-dinitrophenol (DDNP, produced at an astonishing 2,000,000 kg per year in China)^30^. Despite its large-scale use (in Japan, China, America, Germany, and other countries), DDNP has three major disadvantages: (1) it is highly sensitive to impact, friction, and electrostatic discharge, making accidental catastrophic explosions a problem. (2) It is photosensitive and turns dark (through decomposition) when irradiated by light, leading to the instability of product quality^25,26^. (3) DDNP is toxic since nitrophenols have been listed as priority pollutants by the US Environmental Protection Agency and Ministry of Ecology and Environment of China^18,31–33^. As a result, the industrial application of DDNP has long been blamed as a cause of health and environmental hazards as well as criticized for its poor shelf-life and reliability. However, we cannot solve the problems posed by DDNP until new environmentally friendly alternatives are discovered.

**Table 1:** Some representative primary explosives in history

In the light of the aforementioned issues, an enormous challenge remains to find green primary explosives for practical application that exhibit several features and properties including (1) high priming ability; (2) chemical stability for an extended period; (3) appropriate sensitivity to initiation via non-explosive stimulus balanced by relative safety of handling and transport; (4) a lack of sensitivity to moisture and light; (5) thermally stability to at least 150 °C; (6) a lack of toxic metals, perchlorate, and nitrophenols; (7) a reasonably free flowability and resistance to dead-pressing; and (8) low cost and easy preparation^1,2,34^. According to these stringent criteria, there is a lack of suitable examples of green primary explosives thus far.

**Fig. 1:**
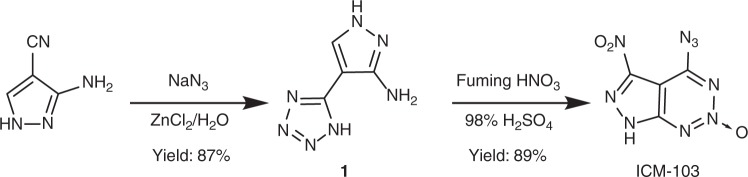
Synthesis of ICM-103. Synthetic pathway for the synthesis of 6-nitro-7-azido-pyrazol[3,4-d][1,2,3]triazine-2-oxide (ICM-103)

Herein we report an organic energetic molecule featuring a fused-ring structure, 6-nitro-7-azido-pyrazol[3,4-d][1,2,3]triazine-2-oxide, (named as ICM-103) which is readily synthesized in high yields through a facile two-step reaction (as Fig. 1). Notably, this energetic compound (ICM-103) for use as a primary explosive affords surprisingly high igniting capability (minimum primary charge (MPC) 60 mg and actual charge (AC) 90–100 mg) and simultaneously exhibits unusual sensitivities towards non-explosive stimuli (i.e., is relatively safe from impact, friction, and electrostatic discharge ignition, yet it is very sensitive to ignition via flame). Moreover, nearly all important properties and performances of this newly discovered initiating substance (ICM-103) surpass those found in the industrially used toxic primary explosive DDNP, thus demonstrating its great promise for use in practical applications.

## Results

### Materials synthesis

In the synthesis of ICM-103 (as Fig. 1), compound **1** is synthesized in a high yield of 87 wt% by the addition of sodium azide to commercially available 3-amino-4-cyan-pyrazole in water with ZnCl_2_ as the catalyst (see Methods, Supplementary Fig. 1). After that, a nitration reaction was followed by treating **1** with a mixture of fuming HNO_3_ and 98% H_2_SO_4_ at 50 °C for 4 h, yielding a water-insoluble light yellow solid (ICM-103) in a high yield of 89 wt%. It is worth noting that, the introduction of azidation (C–N_3_) and nitration (C–NO_2_) as well as the formation of fused-ring 1,2,3-triazine-2-oxide are surprisingly achieved in a single step, which makes the production of ICM-103 highly competitive and allows for further scale-up on a large scale (as Supplementary Fig. 2). Further studies have shown that the reaction is actually a continuous two-step reaction, including cyclization at room temperature and nitrification under heating (as supplementary Fig. 3). Based on the above facts, we also propose a possible mechanism to rationalize the cyclization reaction (as supplementary Fig. 4, Supplementary Tables 1–2).

### Crystal structure

**Fig. 2:**
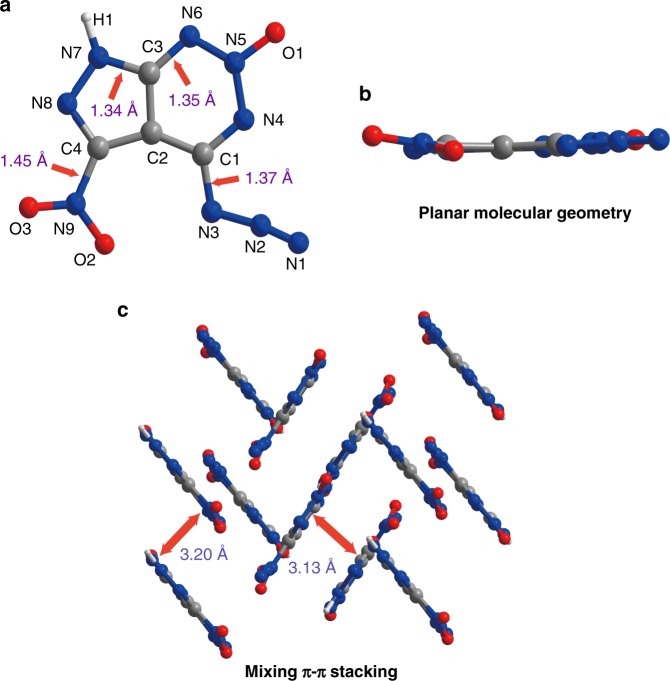
Molecular structure and crystal packing of ICM-103. **a** The molecular structure of ICM-103. **b** The planar molecular geometry of ICM-103. **c** The crystal stacking structure of ICM-103

Single crystals of ICM-103 suitable for X-ray diffraction were obtained via slow crystallization from methanol. The molecule crystallizes in the monoclinic *P*2_1_/*C* space group (as Fig. 2, Supplementary Figs. 5–13 and Supplementary Tables 3–9). The fused-ring shows a nearly flat molecular geometry, and π–π interactions are abundant in the system. The bond-lengths of C–C, C–N, C=N, N–N in the fused-ring are in the range of 1.39–1.40, 1.34–1.35, 1.31–1.33, and 1.34–1.35 Å, respectively. Interestingly, the C–N bond-lengths of C–N_3_ (1.37 Å) and C–NO_2_ (1.45 Å) are slightly longer than the C–N bond-lengths of the fused ring (1.34–1.35 Å), indicating that the C–NO_2_ and C–N_3_ bonds may be responsible for the triggering ignition due to their higher priority of C–N cleavages. Moreover, the crystal packing of this fused-ring molecule shows a mixing π–π stacking mode, which partly accounts for its high sensitivities towards impact and friction^35,36^.

### Physicochemical properties

As is well known, high density structure is conducive to superior energy output and miniaturization of IEDs. Here, the density of ICM-103 measured by gas pycnometry is 1.86 g cm^−3^, which is higher than that of DDNP (1.71 g cm^−3^, see Table 2 and Supplementary Table 10). However, primary explosives are generally prepared in the form of powdery material with low bulk densities. The bulk density of ICM-103 is measured as 0.37 g cm^−3^, which is higher than that of DDNP (0.27 g cm^−3^)^37^. In addition to density considerations, environmental resistance is also an important aspect to consider for primary explosives. It is known that DDNP has the significant drawback of being too photosensitive and that it decomposes rapidly when irradiated by sunlight. As a result, commercial DDNP is a dark substance, due to unknown impurities. This photo-induced decomposition causes continuous metamorphism and seriously erodes initiating efficiency of DDNP. Fortunately, ICM-103 is insensitive to light and moisture and can maintain high purity even after long exposure to sunlight (see Supplementary Fig. 14); thus, relative to DDNP, ICM-103 exhibits strong environmental resistance. As mentioned above, the poor water solubility of ICM-103 was observed in the post-treatment procedure of the nitration reaction. It shows a low solubility in water (0.08 g in 100 mL H_2_O at 25 °C, see Supplementary Table 11) which is slightly lower than that of DDNP (0.10 g under identical conditions) and greatly facilitates the formation of products. For primary explosives, the hygroscopicity has a significant impact on product loading and long-term stability. We further measured the hygroscopicity of ICM-103 by quickly transferring a dry sample to a humidity chamber. After exposure for 24 h, the water content of ICM-103 reached 0.18 wt% (see Supplementary Table 11), much lower than that of DDNP (0.36 wt%), demonstrating that ICM-103 is a non-hygroscopic compound.

**Table 2:** Physical and detonation properties of ICM-103, LA, and DDNP

Items	LA^a^	DDNP^b^	ICM-103^c^
Formula	N_6_Pb	C_6_H_2_N_4_O_5_	C_4_HN_9_O_3_
*M*^d^ (g mol^−1^)	291.3	210.1	223.1
N^e^ (%)	28.9	26.7	56.5
Metal^f^ (%)	71.1	0.0	0.0
Ω_CO_^g^ (%)	−11.0	−15.23	−10.75
*ρ*^h^ (g cm^−3^)	4.80^20^	1.72^37^	1.86
Δ_*f*_*H*_*m*_^i^ (kJ mol^−1^)	450.1^20^	321^37^	744.75
*D*^j^ (km s^−1^)	5920^20^	6900 (1.6 g cm^−3^)^37^	9111
*P*^k^ (GPa)	33.8^20^	24.2	35.14
IS^l^ (J)	2.5–4^20^	1^37^	4
FS^m^ (N)	0.1–1^20^	24.7^37^	60
EDS^n^ (mJ)	<5^20^	1.8^37^	130
FlameS^o^ (cm)	<8	17	>60
*T*_dec_^p^ (°C)	315.0^20^	157^37^	160.3
PD^q^ (MPa)	<118 (or 78)^37^	<60	>100
MPC/AC^r^ (mg)	10/20–30	70/230–280	60/90–100

^a^LA: lead azide

^b^DDNP: 2-diazo-4,6-dinitrophenol

^c^ICM-103: 6-nitro-7-azido-pyrazol[3,4-d][1,2,3]triazine-2-oxide

^d^Formula weight

^e^Nitrogen content

^f^Metal content

^g^Oxygen balance based on CO

^h^Crystal density

^i^Heat of formation

^j^Calculated detonation velocities

^k^Calculated detonation pressure

^l^Impact sensitivity

^m^Friction sensitivity

^n^Electrostatic discharge sensitivity

^o^Flame sensitivity

^p^Decomposition temperature

^q^Pressure durability, below which primary explosives is dead-pressed

^r^Minimum primary charge (MPC) and actual charge (AC)

### Thermal stability

Thermal stability is an important property for primary explosives. In some special cases, the storage and working temperatures of munitions may reach >70 °C^38^. For some ordnance applications, tolerance to higher temperatures are needed, e.g., the operation temperature of a weapon may even exceed 100 °C in the desert^38^. Differential scanning calorimetry (DSC) was used to assess thermal stability of ICM-103. The thermal decomposition of ICM-103 occurs with an onset temperature of 160.3 °C (see Supplementary Figs. 15–16), which is slightly higher than that of DDNP (157 °C), which can satisfy most military and civilian demands. Moreover, the long-term stability was tested under atmospheric pressure at 75 °C for a period of 48 h. Weight loss of ICM-103 was negligible for the sample (<5 mg on an approx. 2 g sample, see Supplementary Table 12) and there were no changes in the appearance or particle morphology visible by optical microscopy, demonstrating that ICM-103 has sufficient long-term thermal stability and is probably capable of passing the most rigorous tests for use in both military and civil applications.

### Sensitivities towards non-explosive stimuli

**Fig. 3:**
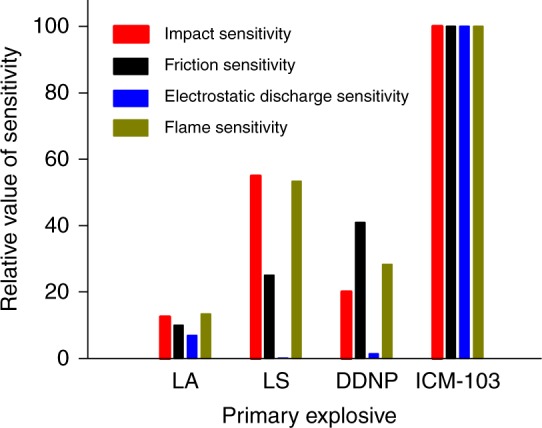
The comparison of sensitivities of several typical primary explosives and ICM-103. Comparison of impact, friction, electrostatic discharge, and flame sensitivities for LA, LS, DDNP, and ICM-103

From the perspective of safety and reliability, the sensitivities of primary explosives to non-explosive stimuli are of particular concern. To assess the hazards and ignition performance involved in the production, transportation, storage, and typical uses/handling of primary explosives, several standards are used to determine the sensitivities of ICM-103 towards impact, friction, electrostatic discharge, and flame (Supplementary Tables 13–20). Among them, sensitivities to impact, friction, and electrostatic discharge are the main parameters that determine the safety of primary explosives, while flame sensitivity is the dominant influence which determines initiation reliability. The higher the measured values of the four sensitivities, the better the safety and reliability. It is clearly seen from the data presented in Table 2 that ICM-103 shows unusual sensitivities towards mechanical stimulus and flame. Specifically, the sensitivities of ICM-103 towards impact, friction, and electrostatic discharge are 4 J, 60 N, and 130 mJ, respectively, all of which are superior to those of DDNP (1 J, 24.7 N, and 1.8 mJ) and LA (2.5–4 J, 0.1–1 N, and <5 mJ) (see Table 2) for practical use; thus the data demonstrate the safety of ICM-103 for use as a primary explosive. Incredibly, the flame sensitivity of ICM-103 was measured as *H*_50_ ≥ 60 cm (*H*_50_ of flame sensitivity reflects the reliability of ignition), which reached the limit of our testing conditions. This result is far better than that of DDNP (*H*_50_ = 17 cm) and LA (*H*_50_ < 8 cm), strongly supporting the observation that ICM-103 exhibits properties of ease of flame ignition substance and that ICM-103 shows excellent ignition reliability. In order to demonstrate the advantages of ICM-103 more clearly, we compared the four sensitivities of ICM-103 with those of the three most widely used primary explosives, LA, LS, and DDNP, based on least normalized sensitivity values (as Fig. 3 and Supplementary Table 21). Based on the high values of ICM-103, it is clear that it exhibits not only superior safety, but also has super-first-class flame sensitivity. The excellent safety properties of ICM-103 are related to the structure of the compound itself, including the well-stabilized fused-ring structure and the large number of π–π interactions which cause an energy-buffering effect, while the hypersensitivity to flame ignition is related to the C–N_3_ and N → O groups. The arked selective sensitivities towards various stimuli make ICM-103 satisfactory both in its safety characteristics and in its reliability for ignition via flame.

### Detonation performances

To investigate the energetic properties of ICM-103, its detonation performances were studied. According to the method developed by Byrd and Rice^39,40^, the heat of formation of ICM-103 was calculated as 744.75 kJ mol^−1^ (see Supplementary Fig. 17, Supplementary Note 1, Supplementary Equations 1–2), which is close to that measured via heat of combustion experiments (739.40 kJ mol^−1^; see Supplementary Note 2, Supplementary Equations 3–5). Using the measured density and calculated heat of formation, the detonation velocity (*D*) and pressure (*P*) of ICM-103 were calculated as 9111 m s^−1^ and 35.1 GPa (see Supplementary Table 22, Supplementary Fig. 18), respectively (based on EXPLO5 version 6.02). To our knowledge, both the *D* and *P* values of ICM-103 are the highest ones known among all the inorganic and organic primary explosives; they are even comparable to the famous powerful secondary explosives RDX (1,3,5-trinitro-1,3,5-triazacyclohexane) and HMX (1,3,5,7-tetranitro-1,3,5,7-tetrazocine)^41^. Considering that high detonation performances will facilitate the integration of primary explosives and main charge, we envisage that ICM-103 may simplify the charge structure of primers and detonators (for details see Supplementary Fig. 19), suggesting that ICM-103 is well suited for practical use in both military and civil applications. Subsequently, we evaluated the detonation properties of ICM-103 through the reported method using LA as the primary explosive^24^. In the detonation test, 60 mg LA was used as primary explosive to detonate 500 mg RDX or 500 mg ICM-103 with a pyrotechnical igniter. Both RDX and ICM-103 as secondary explosives can be initiated successfully using the LA as primary explosive (Supplementary Fig. 20). The aperture of perforated lead plate in the test of using RDX and ICM-103 separately is ca. 1.3 and 1.5 cm, respectively, demonstrating that ICM-103 shows the higher detonation performances than that of RDX.

### Initiating efficiency

**Fig. 4:**
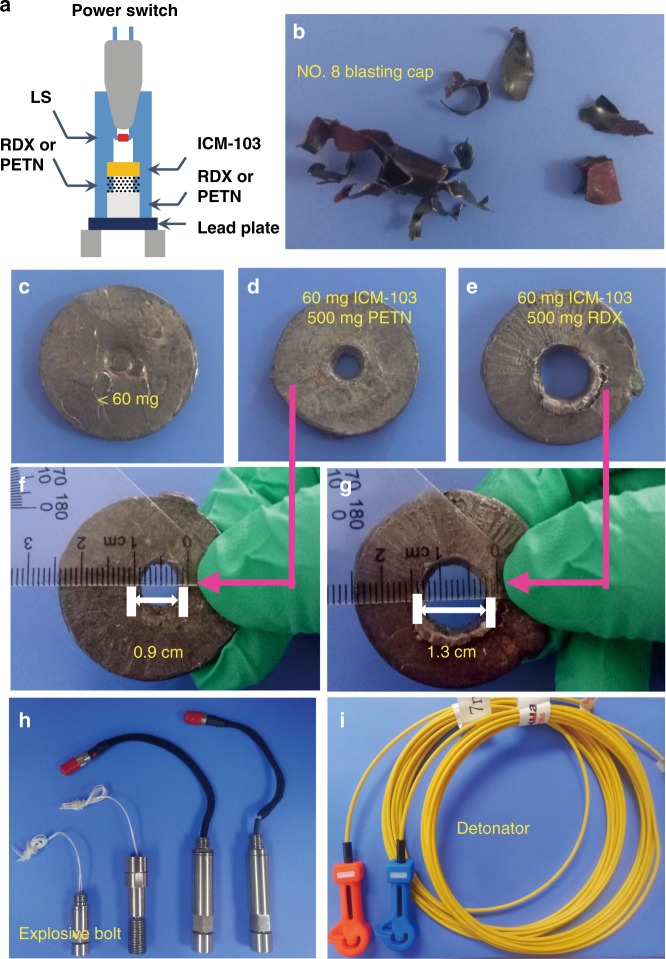
Initiation capability test of ICM-103. **a** Initiation capability test apparatus. **b** Fragmentation of the cap caused by a deflagration-to-detonation transition (DDT) of PETN or RDX. **c** One of the lead plates that cannot be blasted out of the hole by using <60 mg ICM-103 sample. **d**–**g** Lead plates that were blasted out of the hole by using 60 mg ICM-103 sample, with PETN and RDX as main charge, respectively. **h**, **i** Two important products charged with 60–80 mg ICM-103 samples

The initiating efficiency, perhaps the most important parameter, determines the ability of a primary explosive to initiate secondary explosives. In this study, the igniting efficiency of ICM-103 was evaluated by the MPC, which was conducted by the detonation against a lead plate (thickness: 5 mm) using pentaerythrite tetranitrate (PETN) or 1,3,5-trinitro-1,3,5-triazacyclohexane (RDX) as a secondary explosive and defined by a critical weight, below which the lead plate cannot be blasted out of the hole. In this MPC test, a certain amount of ICM-103 is filled in a No. 8 blasting cap and pressed with a static pressure of 32 MPa, and then it is fired by a standard pyrotechnical igniter (see Fig. 4). The test results demonstrate that 60 mg ICM-103 can detonate both PETN and RDX to bore through the lead plate. The priming ability of ICM-103 (MPC = 60 mg) is superior to that of DDNP (MPC = 70 mg). However, due to the poor photochemical stability and continuous metamorphism in the storage process, the AC of DDNP in IEDs must be at least 3 times (i.e., ≥230 mg) of its MPC to ensure the initiating reliability. By contrast, ICM-103 shows good environmental resistance and thus avoids the metamorphism problem. As a result, its AC is decreased to around 90–100 mg. This is a great advantage of ICM-103 than DDNP for real applications, especially in special military fields like sophisticated weapons and advanced manufacturing industries.

### The flowability and pressure durability

The flowability of a primary explosive directly influences its charge and mixing properties. To ensure that the samples can be smoothly loaded into the detonators in an actual production environment, it must have satisfactory flowability, which is often assessed by the angle of repose (*θ*). The smaller the *θ*, the better the processability of the sample. Using a standard method and a given device, the *θ* of ICM-103 was measured to be 28.76°, which is almost equivalent to that of DDNP (*θ* = 29.01°)^42^, thus demonstrating satisfactory flowability of ICM-103. In addition to high initiating ability and high charge density, the primary explosives filled in detonators must be pressed under a certain pressure, but in this case some primary explosives are found to be dead-pressed or even explosions. As expected, ICM-103 exhibited a surprisingly high pressure durability (PD) of >100 MPa, greater than that of DDNP (<60 MPa)^43^, suggesting the good PD and reliability of ICM-103. We have successfully applied ICM-103 to charge two commonly used IEDs, i.e., the explosive bolt and the detonator (Fig. 4h, i), and both IEDs were assembled according to established industrial methods. Both IEDs showed excellent initiating performances and reliability at a given charge level of 90–100 mg, demonstrating the great promise of ICM-103 in commercial IEDs.

## Discussion

In conclusion, we report the facile synthesis of an organic primary explosive ICM-103 from commercially available reagents. Its structure has been determined by single-crystal X-ray diffraction and shows a nearly flat molecular geometry. It exhibits a high measured density, good thermal stability, positive heat of formation, and excellent detonation performances. From the perspective of real-world applications, ICM-103 meets all the industrially desired criteria for green primary explosives, including low-cost production and easy production scalability, high priming ability, good environmental resistance, satisfactory mechanical and flame sensitivities, and reasonable flowability and PD; in addition, it is free of toxic metals, perchlorate, nitrophenol, etc. It can be considered that nearly all important properties and performances of ICM-103 are superior to those of DDNP, the most widely used primary explosive in industry. ICM-103 thus demonstrates great promise as a drop-in DDNP replacement for commercial IEDs. Given the undoubted importance of green primary explosives in both military and civil applications, the discovery of ICM-103 may be a significant breakthrough in the field of energetic materials.

## Methods

### General

Caution**!** The compound ICM-103 is a highly energetic material and tends to explode under physical stress. Laboratories and personnel must be properly grounded, and safety equipment such as protective gloves and coats, face shield and explosion-proof baffle are recommended.

### Materials

Sodium azide (99%), 3-amino-4-cyano-pyrazole (98%), and zinc chloride (99%) were purchased from Aladdin. Other commercial reagents were used as received.

### Product characterization

Infrared spectra (IR) is recorded on a Bruker Equinox 55 infrared spectrometer. Elemental analyses (C, H, O, and N) are performed on a varioMICRO cube fully automatic trace element analyzer. ^1^H and ^13^C NMR spectra are recorded on Bruker Advance 400 nuclear magnetic resonance spectrometers. The single crystal X-ray diffraction data collections are carried out on a Rigaku AFC-10/Saturn 724+CCD diffractometer. The density at 25 °C is determined by Micromeritics Accupyc II 1340 pycnometer automatic true density meter. Thermal decomposition temperatures are determined by using DSC and thermogravimetric (TG) on a CDR-4 of Shanghai Precision & Scientific Instrument Co., Ltd. The long-term thermal stability, flame and electrostatic discharge sensitivity, bulk density, flowability, PD, solubility, and MPC test (or initiation capability test) are measured according to the method given by GJB 5891-2006, respectively. Impact and friction sensitivity measurements were made using a standard BAM Fall hammer and a BAM friction tester. The heat of formation is calculated with the Gaussian 09 software. The detonation parameters are calculated based on the program suite of EXPLO5 (version 6.02).

### Synthesis of compound 1

3-amino-4-cyano-pyrazole (1.08 g, 10 mmol), sodium azide (0.75 g, 11.5 mmol), and zinc chloride (1.36 g, 10 mmol) were suspended in water (100 mL) and the mixture reacted under reflux for 12 h. After cooling to room temperature, HCl (2 M, 7.5 mL) was added dropwise to the reaction mixture. The resulting precipitate was filtered, washed with water (3 × 20 mL) and dried yielding **1** (1.31 g, 87%). DSC (10 °C min^−1^, °C): 277 (dec.); IR (KBr pellet, cm^−1^): 3434 (s), 3381 (m), 3216 (s), 2936 (m), 2761 (m), 2350 (w), 1623 (s), 1533 (w), 1408 (m), 1264 (m), 1049 (w), 751 (m), 593 (m). ^1^H NMR (400 MHz, DMSO-d_6_, 25 °C): δ = 14.54 (br, 1H, NH), 8.40 (s, 2H, NH_2_) ppm; ^13^C NMR (100 MHz, DMSO-d_6_, 25 °C): δ = 157.99, 156.35, 133.05, 96.46 ppm; EA calcd for C_4_H_5_N_7_ (151.13 g mol^−1^): C 31.79, H 3.33, N 64.88%; Found: C 31.77, H 3.34, N 64.91%.

### Synthesis of ICM-103

0.5 g compound **1** was added with portion to a stirred mixture of 98% H_2_SO_4_ (10 mL) and fuming HNO_3_(10 mL), while maintaining the reaction temperature below 0 °C. After the addition was complete, the ice bath was removed, and the mixture was allowed to warm slowly to 50 °C. It was stirred for another 4 h, and then the reaction mixture was poured into crushed ice (20 g). The precipitate was filtered, washed with cold water (2 × 10 mL), and dried in vacuum to yield the target compound ICM-103 as a light yellow solid. Yield: 0.65 g, 89%. DSC (10 °C min^−1^, °C): 160.3 (dec.); IR (KBr pellet, cm^−1^): 3442 (s), 2244 (w), 2204 (w), 2155 (m), 1598 (s), 1535 (s), 1371 (s), 1341 (m), 1209 (m), 871 (m), 827 (m), 740 (s), 688 (m), 540 (w). ^1^H NMR (400 MHz, DMSO-d_6_, 25 °C): δ = 14.49 ppm (br, 1H, NH); ^13^C NMR (100 MHz, DMSO-d_6_, 25 °C): δ = 157.95, 157.31, 148.07, 90.03 ppm; EA calcd for C_4_HN_9_O_3_ (223.1 g mol^−1^): C 21.53, H 0.45, N 56.50, O 21.51%; Found: C 21.17, H 0.40, N 56.31, O 21.29%.

## Supplementary information

Supplementary Information

## Data Availability

The data that support the findings of this study are available from the corresponding authors on request. The X-ray crystallographic coordinates for structures reported in this study have been deposited at the Cambridge Crystallographic Data Centre (CCDC), under deposition numbers 1850200 (**1**) and 1842901 (ICM-103). These data can be obtained free of charge from The Cambridge Crystallographic Data Centre via www.ccdc.cam.ac.uk/data_request/cif.
