# Supplementary material for: A green metal-free fused-ring initiating substance
Source: Nat Commun. 2019 Mar 22;10:1339. doi: 10.1038/s41467-019-09347-y (PMC6430815; doi:10.1038/s41467-019-09347-y)
Supplement: Supplementary file 1 — Supplementary Information [file 41467_2019_9347_MOESM1_ESM.pdf]

Supplementary Information

A green metal-free fused-ring initiating substance

Deng and Feng *et. al.*

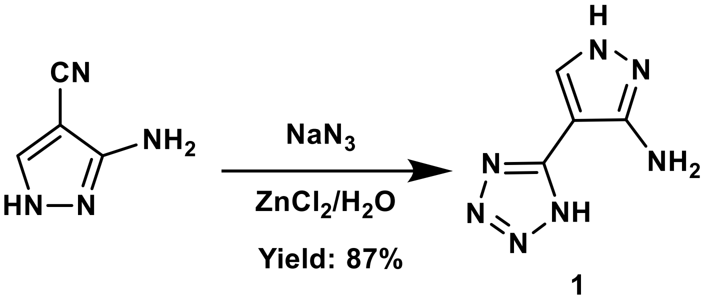

Supplementary Figure 1. The synthesis of compound 1.

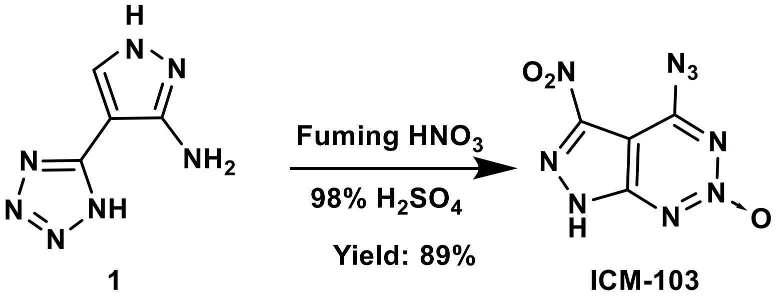

Supplementary Figure 2. The synthesis of ICM-103.

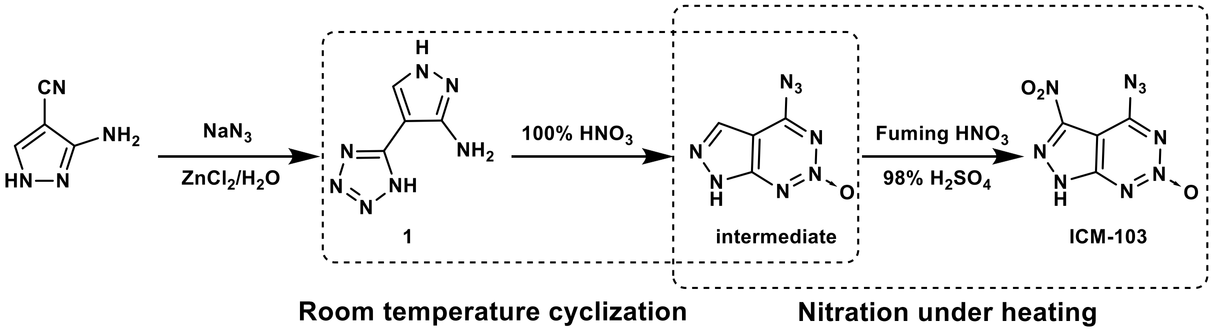

Supplementary Figure 3. Two-step reaction process to get ICM-103. We carefully studied the separated products at the room temperature and heating stages. It was found that the reaction was actually a continuous two-step reaction, including cyclization at room temperature and nitrification under heating. The crystal structure was also obtained (CCDC: 1843024).

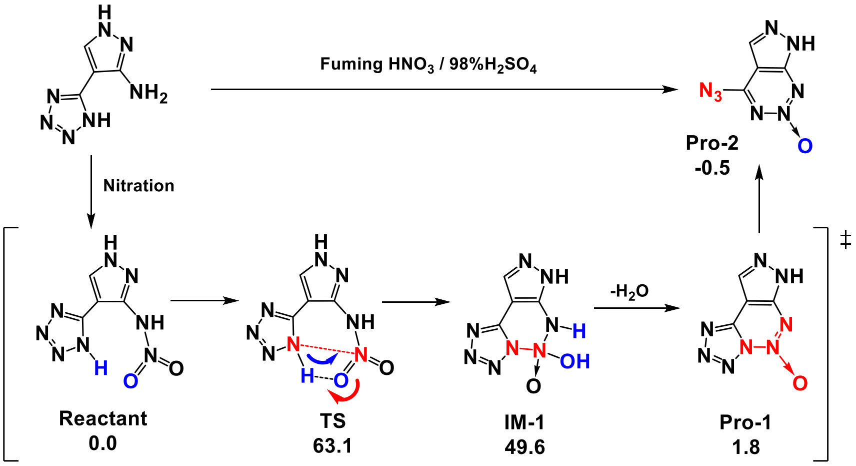

Supplementary Figure 4. The possible mechanism for the formation of ICM-103. Gibbs Free Energy changes at the level of MP2/6-311++G(2df,2p) //B3LYP/6-31+G(d, p) are shown in kcal/mol. In this mechanism, the intermediate product first reacts with the mixed acid to generate an unstable nitramine compound, named Reactant, of which the Gibbs Free Energy is defined as 0 kcal mol^{-1}. Then, induced by hydrogen bond, the electrophilic addition reaction, through a transition state TS with high activation energy barrier (ΔG=+63.1 kcal mol^{-1}), occurs between N-H of tetrazole unit and N=O of nitro group, leading to the ring closure and the formation of an unstable fused ring intermediate IM-1 (ΔG=+49.6 kcal mol^{-1}), which then quickly suffers an elimination reaction of dehydration and form a new fused compound Pro-1 (ΔG=+1.8 kcal mol^{-1}). According to the work of Huynh et al [1], we think Pro-1 is likely to undergo an azido-tetrazolo tautomerizations at high temperatures and irreversibly transform to the final product Pro-2 (ΔG= -0.5 kcal mol^{-1}) at room temperature. In 2015 and 2017, the phenomenon of azido-tetrazolo tautomerizations has been further confirmed by using X-ray single diffraction [2,3].

Supplementary Table 1. The calculated results of the reaction mechanism(structure)

Involved species	Optimized geometry	Involved Species	Optimized geometry
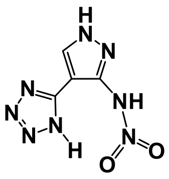	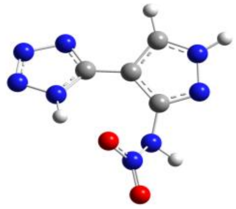	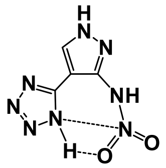	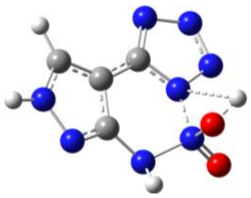
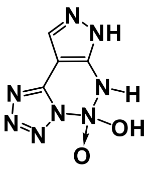	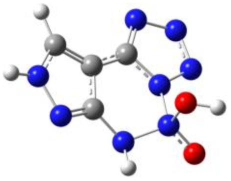	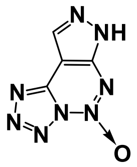	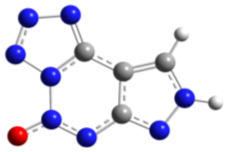
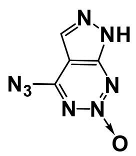	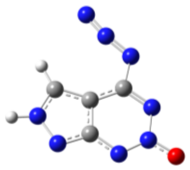		

Supplementary Table 2. The calculated results of the reaction mechanism(energy) All the calculations were performed with Gaussian09 package [4]. The geometry optimizations of all species in this study were performed using B3LYP method [5,6]. The 6-31+G(d,p) basis set was used for all the atoms. Frequency calculations at the B3LYP/6-31+G(d,p) level of theory were carried out to characterize each stationary point (minimum or transition state) and to obtain the thermodynamic corrections to Gibbs free energy. Intrinsic reaction coordinate (IRC) was calculated to confirm the connection between the transition state and the right reactant/product. All the single-point calculations were performed with MP2/6-311++G(2df,2p). The reported Gibbs free energy in this study was in gas phase of 298.15K, 1atm.

Species	E(MP2) (a.u.)	TCG (a.u.)	G(MP2) (a.u.)
Reactant	-741.8262587	0.081100	-741.7451587
TS	-741.7254481	0.080819	-741.6446291
Im-1	-741.7369228	0.080797	-741.6561258
Pro-1	-665.4877002	0.056639	-665.4310612
Pro-2	-665.4883684	0.053580	-665.4347884

The reported Gibbs free energy of all species in this study was calculated in the gas-phase of 298 K.

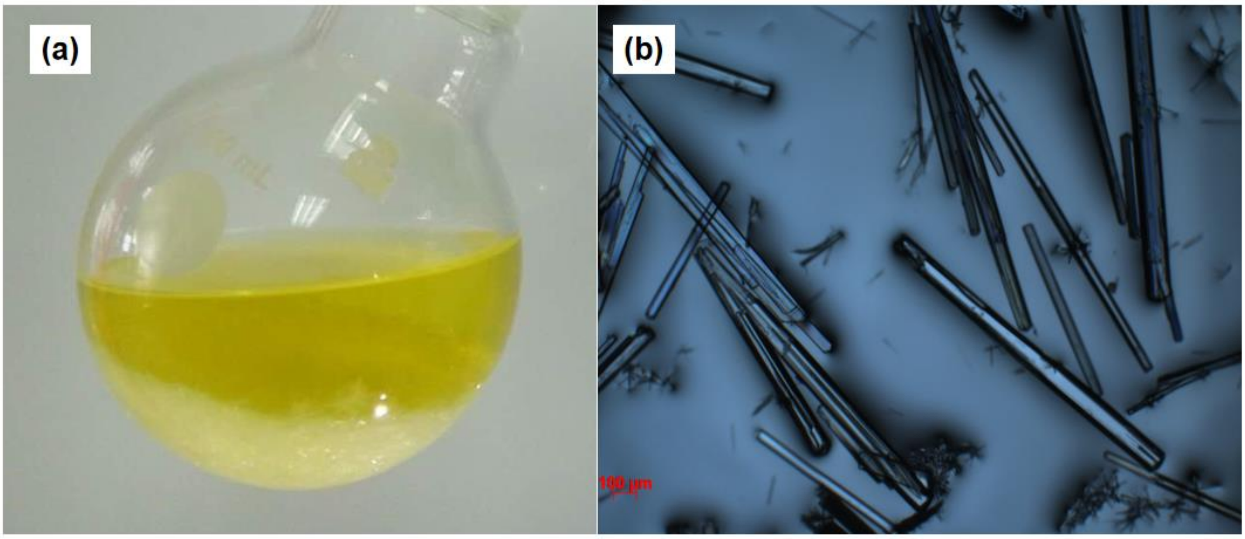

Supplementary Figure 5. a) The crystal products of **ICM-103** recrystallized from methanol; b) the crystal morphology of **ICM-103** observed by optical microscope (100 μm).

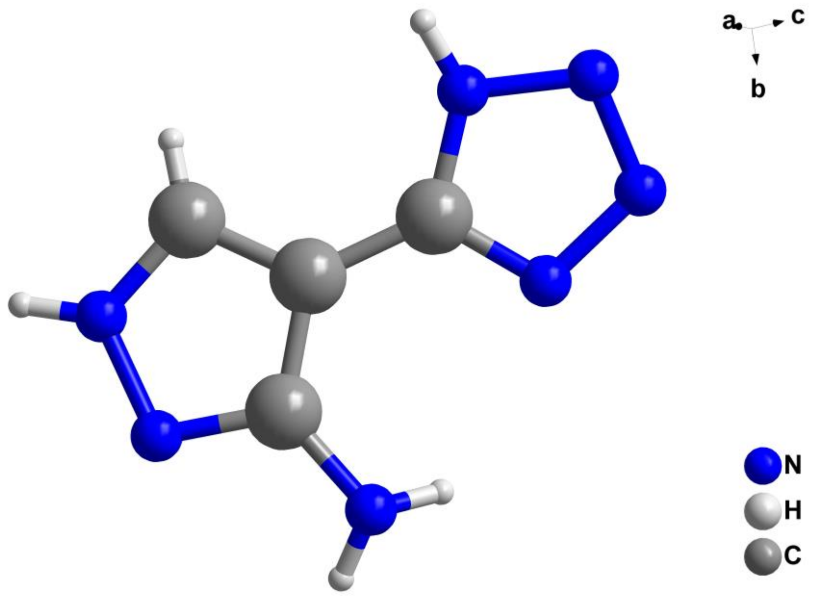

Supplementary Figure 6. The molecule structure of compound **1**.

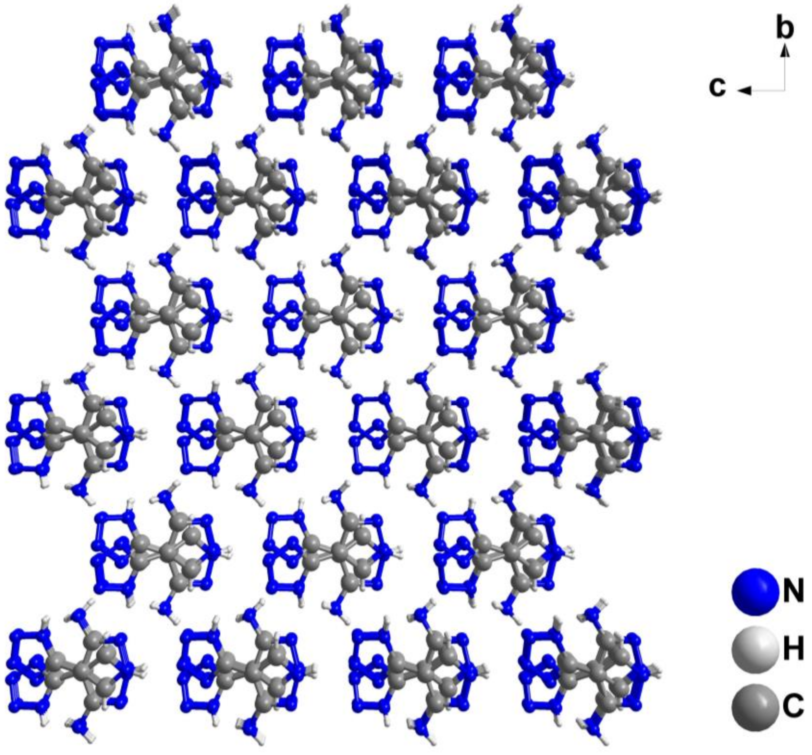

Supplementary Figure 7. The crystal stacking structure of compound **1** seen from a axis.

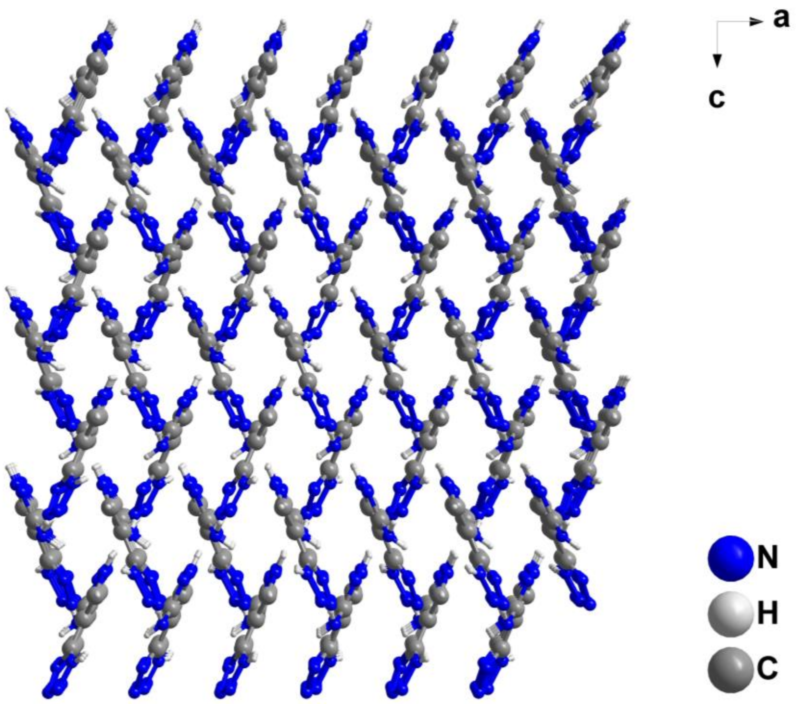

Supplementary Figure 8. The crystal stacking structure of compound **1** seen from b axis.

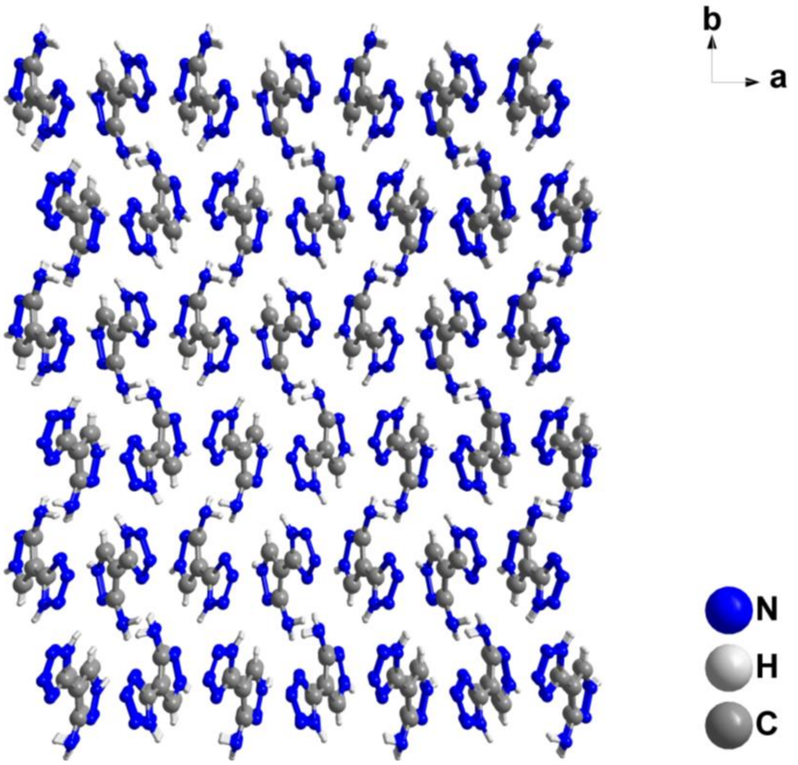

Supplementary Figure 9. The crystal stacking structure of compound **1** seen from c axis.

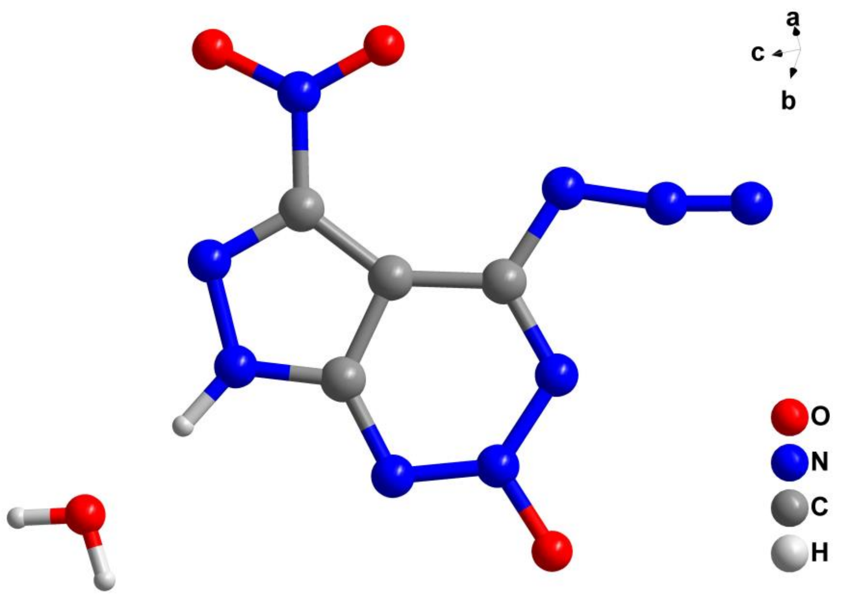

Supplementary Figure 10. The molecule structure of **ICM-103·H_2O**.

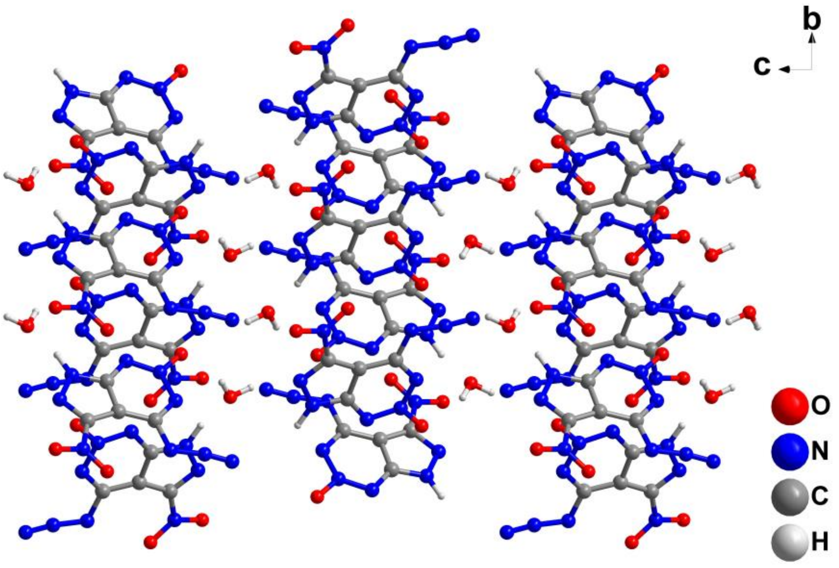

Supplementary Figure 11. The crystal stacking structure of **ICM-103·H_2O** seen from a axis.

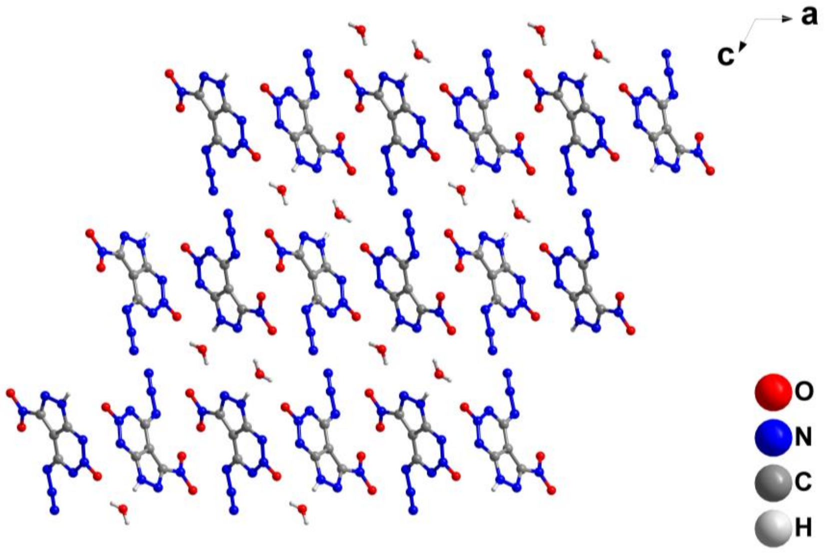

Supplementary Figure 12. The crystal stacking structure of **ICM-103·H_2O** seen from b axis.

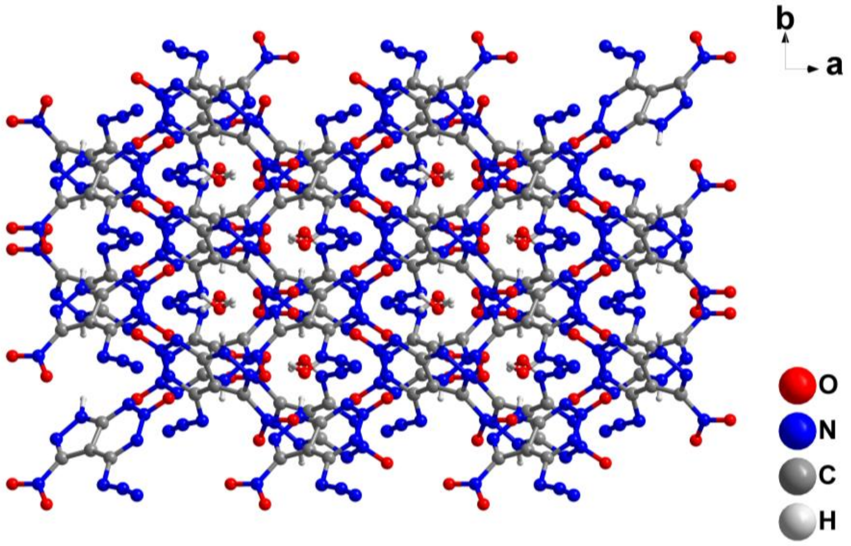

Supplementary Figure 13. The crystal stacking structure of ICM-103·H_2O seen from c axis.

Supplementary Table 3. Crystal data and details of the structure determination

Compound Name	**ICM-103·H_2O**	**1**
Formula	$C_4HN_9O_3\cdot H_2O$	$C_4H_5N_7$
Formula Weight	241.15	151.15
Crystal System	Monoclinic	Orthorhombic
Space Group	P21/c (No.14)	Pna21 (No.33)

a, b, c [Å]	9.931(5), 5.210(3), 19.767(7)	7.469(2) , 10.695(3), 7.726(2)
α, β, γ [θ]	90, 116.419(19), 90	90, 90, 90
V [Å^3]	915.9(8)	617.2(3)
Z	4	4
ρ [g·cm^{-3}]	1.749	1.627
Mu (MoKa) [mm^{-1}]	0.155	0.121
F(000)	488	312
Crystal Size [mm]	0.15 x 0.15 x 0.30	0.30 x 0.30 x 0.30
Temperature (K)	173	173
Radiation [Å]	MoKa 0.71073	MoKa 0.71073
θ [°]	2.3 to 25.0	3.3 to 26.8
Dataset	-11 ≤ h ≤ 11; -6 ≤ k ≤ 6; -23 ≤ l ≤ 23	-9 ≤ h ≤ 8; -11 ≤ k ≤ 13; -9 ≤ l ≤ 9
Tot., Uniq. Data, R(int)	4926, 1568, 0.090	3858, 1305, 0.043
Observed data [I > 2.0 sigma(I)]	1021	1179
N_{ref}, N_{par}	1568, 161	1305, 100
R, wR_2, S	0.0705, 0.2058, 1.01	0.0418, 0.1173, 1.12
Largest diff. peak and hole [e·Å^{-3}]	0.34 / -0.42	0.23 / -0.27

Supplementary Table 4. Bond lengths for compound 1.

Bond	Lengths [Å]	Bond	Lengths [Å]	Bond	Lengths [Å]
N1-N2	1.347(4)	N1-H1	0.8400	N7-H7A	0.8400
N1-C1	1.332(4)	N5-H5	0.8400	N7-H7B	0.8400
N2-N3	1.286(5)	N5-C4	1.333(4)	C1-C2	1.445(4)
N3-N4	1.354(4)	N6-C3	1.338(4)	C2-C3	1.421(5)
N4-C1	1.330(4)	N7-C3	1.371(4)	C2-C4	1.390(4)
N5-N6	1.372(4)	C4-H4	0.8400		

Supplementary Table 5. Bond angles for compound 1.

Bond	Angle [°]	Bond	Angle [°]	Bond	Angle [°]
N2-N1-C1	109.7(3)	N4-C1-C2	124.3(3)	N6-C3-N7	121.1(3)
N2-N3-N4	111.3(3)	N1-C1-C2	128.4(3)	N6-C3-C2	110.9(3)
N6-N5-C4	113.0(2)	C1-C2-C3	125.5(3)	N7-C3-C2	128.0(3)
N5-N6-C3	104.4(3)	C3-C2-C4	104.9(3)	N5-C4-C2	106.8(3)
C3-N7-H7A	110.00				

Supplementary Table 6. Torsion angles for compound 1.

Bond	Torsion angle [°]	Bond	Torsion angle [°]	Bond	Torsion angle [°]
C1-N1-N2-N3	0.2(4)	N5-N6-C3-N7	178.4(3)	C1-C2-C3-N6	-179.1(3)
N2-N1-C1-C2	-179.9(3)	N5-N6-C3-C2	0.8(3)	C4-C2-C3-N6	-0.7(4)
N2-N3-N4-C1	0.6(4)	N1-C1-C2-C3	-171.8(3)	C1-C2-C4-N5	178.6(3)
N3-N4-C1-C2	179.7(3)	N4-C1-C2-C3	8.0(5)	C3-C2-C4-N5	0.3(4)
N6-N5-C4-C2	0.2(4)				

Supplementary Table 7. Bond lengths for ICM-103·H_2O.

Bond	Lengths [Å]	Bond	Lengths [Å]	Bond	Lengths [Å]
O1-N5	1.253(5)	N3-C1	1.372(5)	N8-C4	1.313(5)
O2-N9	1.214(5)	N4C1	1.334(5)	N9-C4	1.447(6)
O3-N9	1.222(5)	N4-N5	1.346(5)	N7-H7	0.98(5)
O4-H4A	0.8500	N5-N6	1.309(4)	C1-C2	1.403(5)
O4-H4B	0.8500	N6-C3	1.352(6)	C2-C3	1.393(6)
N1-N2	1.050(7)	N7-N8	1.352(6)	C2-C4	1.399(6)

Supplementary Table 8. Bond angles for ICM-103·H_2O.

Bond	Angle[°]	Bond	Angle[°]	Bond	Angle[°]
H4A-O4-H4B	105.00	O2-N9-O3	124.8(4)	C3-C2-C4	102.5(3)
N1-N2-N3	171.4(6)	O3-N9-C4	118.4(4)	N6-C3-N7	124.0(4)
N2-N3-C1	114.5(4)	C3-N7-H7	125(3)	N8-C4-N9	118.4(4)
O1-N5-N4	114.4(3)	N3-C1-N4	121.0(4)	N9-C4-C2	128.9(4)
N8-N7-C3	110.7(3)	C1-C2-C3	113.5(4)		

Supplementary Table 9. Torsion angles for ICM-103·H_2O.

Bond	Torsion angle [°]	Bond	Torsion angle [°]	Bond	Torsion angle [°]
N2-N3-C1-C2	-177.9(4)	C3-N7-N8-C4	0.0(5)	N4-C1-C2-C3	0.5(6)
C1-N4-N5-O1	177.8(4)	N8-N7-C3-C2	1.1(5)	N5-N4-C1-C2	0.1(6)
N5-N4-C1-N3	178.2(4)	N7-N8-C4-N9	178.7(4)	O2-N9-C4-C2	-11.2(7)
O1-N5-N6-C3	-177.2(4)	N5-N6-C3-N7	177.7(4)		

Supplementary Table 10. The density of ICM-103.

Items	No.1	No.2	No.3	No.4	No.5	No.6	No.7	No.8	No.9	No.10	Average
Actual density [$g \cdot cm^{-3}$] [a]	1.853	1.872	1.861	1.862	1.862	1.862	1.871	1.863	1.855	1.859	1.862
Bulk density [$g \cdot cm^{-3}$] [b]	0.343	0.343	0.347	0.344	0.343	0.344	0.346	0.343	0.345	0.344	0.344

[a] Density measured by gas pycnometer (25 ^{o}C). [b] Apparent density measured at room temperature (25 ^{o}C).

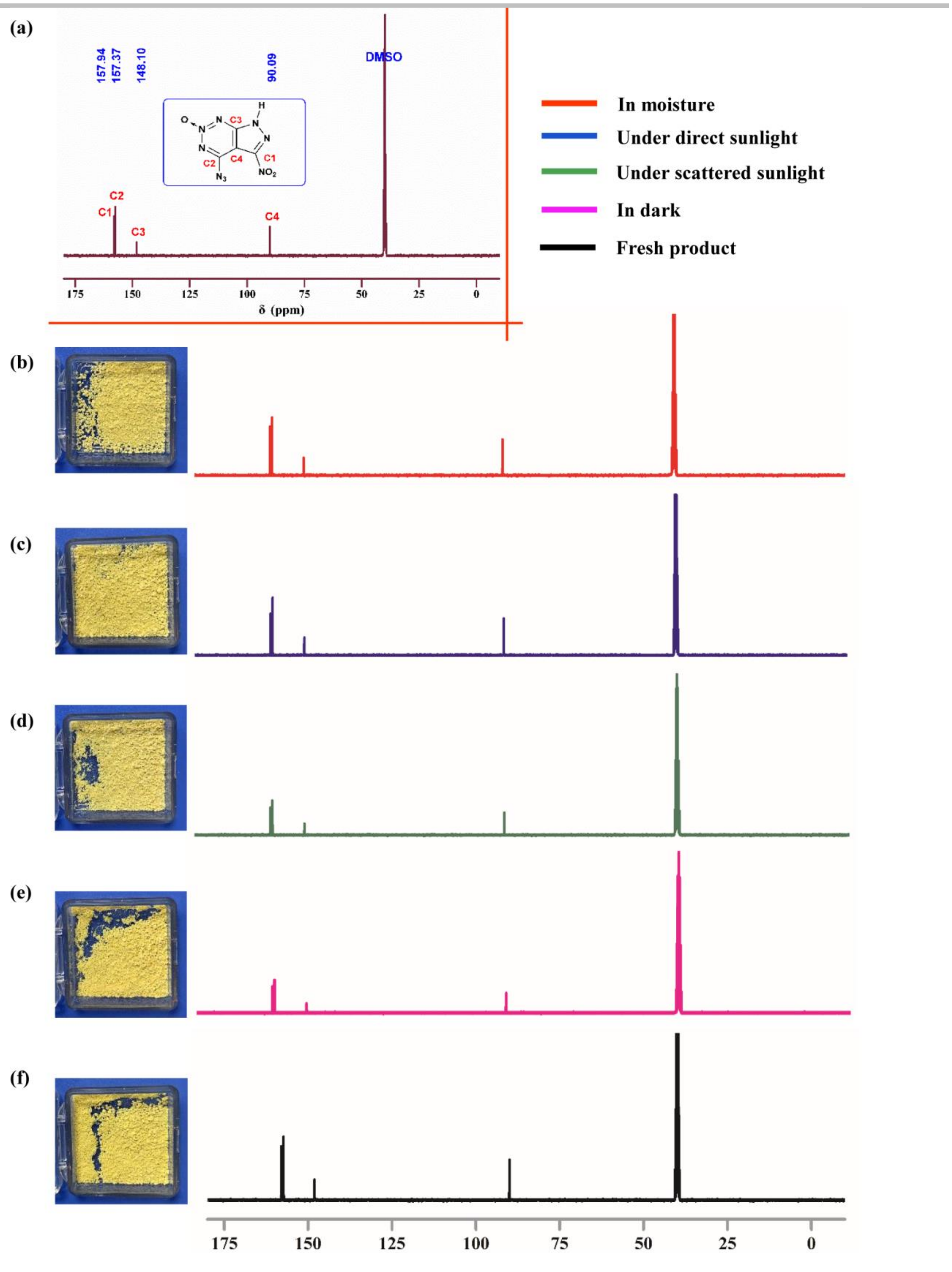

Supplementary Figure 14. Environmental resistance. (a) ^{13}C NMR of ICM-103; (b) ^{13}C NMR of ICM-103 measured after 48 hours stand in moisture; (c) ^{13}C NMR of ICM-103 measured after 2 hours of direct sunlight; (d) ^{13}C NMR of ICM-103 measured after 12 hours of scattered sunlight; (e) ^{13}C NMR of ICM-103 measured after 48 hours stand in dark; (e) ^{13}C NMR of fresh ICM-103.

Supplementary Table 11. The solubility and hygroscopicity of ICM-103.

Items	No.1	No.2	No.3	No.4	No.5	No.6	No.7	No.8	No.9	No.10	Average
Solubility [g·(100mL)$^{-1}$] [a]	0.06	0.07	0.10	0.08	0.08	0.07	0.09	0.07	0.08	0.09	0.08
Hygroscopicity [mg] [b]	4.8	5.41	5.43	5.41	5.12	5.42	5.42	5.72	5.71	5.43	5.39
Hygroscopicity [%] [b]	0.16	0.18	0.18	0.18	0.17	0.18	0.18	0.19	0.19	0.18	0.18

[a] The quality of the tested sample is 0.5 g and the solution is H_2O. [b] The quality of the tested sample is 3 g.

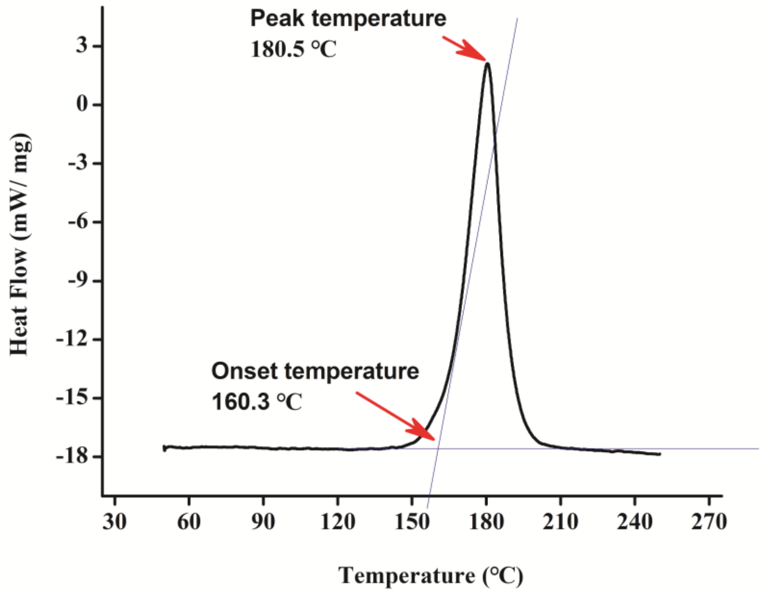

Supplementary Figure 15. Thermal decomposition of ICM-103. DSC curve of ICM-103 (The powder is dried at 70 ℃ for 2 hours before test). The thermal decomposition behavior of the compound ICM-103 is investigated by differential scanning calorimetry (DSC) and Thermogravimetric (TG) at a heating rate of 10 ℃·min^{-1}. As shown in Supplementary Figures 15 and 16, the decomposition of ICM-103 occurs with an onset temperature at 160.3 ℃ and a peak temperature at 180.5 ℃, which is high than the criterion of 150 ℃ for green primary explosives.

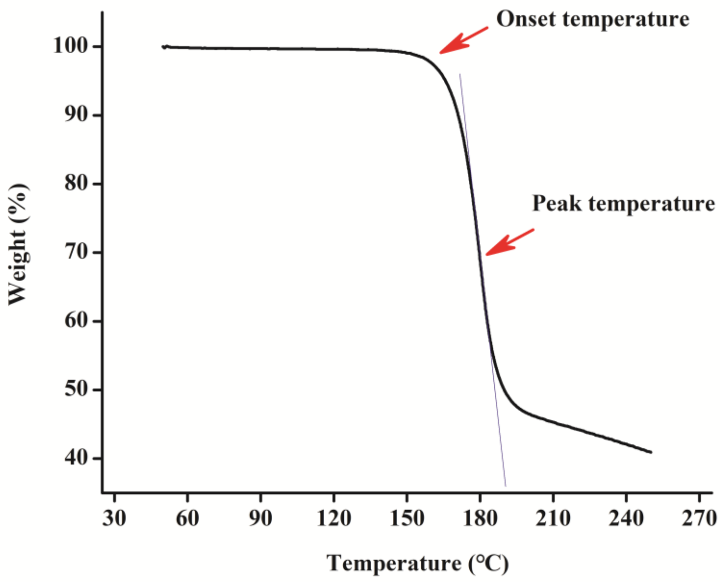

Supplementary Figure 16. TG curve of ICM-103 (The powder is dried at 70 °C for 2 hours before test)

Supplementary Table 12. The results of the long-term thermal stability testing of ICM-103. The long-term thermal stability is tested by the following device at 75 °C for 48 hours.

	No.1	No.2	No.3	No.4	No.5	No.6	No.7	No.8	No.9	No.10	Average
w [g] [a]	0.09	0.07	0.10	0.08	0.08	0.07	0.06	0.07	0.08	0.09	0.08

[a] The quality of the sample after heating.

Supplementary Table 13. The record of flame sensitivity test (fuse method). 0.020 g ± 0.005 g of the powders subjected to high-temperature drying treatment(50 °C, 4 h) are weighed and placed in a sample crucible. The test specimen is pressed with a press at a pressure of 58.8 MPa, and 40 shots are pressed and stored in a desiccator for use.

i	x_i	1	2	3	4	5	6	7	8	9	10	11	12	13	14
2	0.903			1	1	1	1	1	1	1	1	1	1	1	1
1	0.845		1												
0	0.778	1													
-1	0.699														
-2	0.602														

The result is recorded as “1” for “response” and “0” for “not responding”.

Supplementary Table 14. The record of flame sensitivity test (black powder column method)

i	x_i	1	2	3	4	5	6	7	8	9	10	11	12	13	14
2	1.778			1	1	1	1	1	1	1	1	1	1	1	1
1	1.699		1												
0	1.602	1													
-1	1.477														
-2	1.301														

The result is recorded as “1” for “response” and “0” for “not responding”.

Supplementary Table 15. The record of impact sensitivity test. 0.020 g ± 0.005 g of the powders subjected to high-temperature drying treatment (60 ℃, 2 h) are weighed and placed in a sample crucible. The test specimen is pressed with a screw press at a pressure of 39.2 MPa, and 40 shots are pressed and stored in a desiccator for use.

i	x_i	1	2	3	4	5	6	7	8	9	10	11	12	13	14	15
2	1.653															
1	1.633										1				1	
0	1.613	1		1				1		0		1		0		0
-1	1.591		0		1		0		0				0			
-2	1.568					0										

The result is recorded as “1” for “response” and “0” for “not responding”.

Supplementary Table 16. Continued of the record of impact sensitivity test

i	x_i	16	17	18	19	20	21	22	23	24	25	26	27	28	29	30	31
2	1.653																
1	1.633	1		1		1		1		1		1		1		1	
0	1.613		0		0		0		0		0		0		0		0
-1	1.591																
-2	1.568																

The result is recorded as “1” for “response” and “0” for “not responding”.

Supplementary Table 17. The record of friction sensitivity test. Weighting 20-30 mg of the high temperature treated (50 ℃, 4 h) sample with a quantitative spoon, and placing it on ceramic test piece.

i	x_i	1	2	3	4	5	6	7	8	9	10	11	12	13	14	15
2	1.857					1										
1	1.806				0		1									
0	1.778	1		0				1		1				1		1
-1	1.748		0						0		1		0		0	
-2	1.732											0				

The result is recorded as “1” for “response” and “0” for “not responding”.

Supplementary Table 18. Continued of the record of friction sensitivity test

i	x_i	16	17	18	19	20	21	22	23	24	25	26	27	28	29	30
2	1.857															
1	1.806		1		1		1		1		1		1		1	
0	1.778	0		0		0		0		0		0		0		0
-1	1.748															
-2	1.732															

The result is recorded as “1” for “response” and “0” for “not responding”.

Supplementary Table 19. The record of electrostatic spark sensitivity test. Weighting 20-30 mg of the high temperature treated (50 ℃, 4 h) sample with a quantitative spoon, and placing it on the shot column, and storing it in a desiccator for use.

i	x_i	1	2	3	4	5	6	7	8	9	10	11	12	13	14	15
-2	0.477															
-1	0.602							0		0				0		
0	0.699		0				1		1		0		1		0	
1	0.778	1		0		1						1				1
2	0.845				1											

The result is recorded as “1” for “response” and “0” for “not responding”.

Supplementary Table 20. Continued of electrostatic spark sensitivity test

i	x_i	16	17	18	19	20	21	22	23	24	25	26	27	28	29	30
-2	0.477							0								
-1	0.602		0				1		0		0					
0	0.699	1		0		1				1		0				0
1	0.778				1								0		1	
2	0.845													1		

The result is recorded as “1” for “response” and “0” for “not responding”.

Supplementary Table 21. The sensitivities of several classical primary explosives and ICM-103

Items	T_{dec} (°C) [a]	IS (J) [b]	FS (N) [c]	EDS (mJ) [d]	FlS (cm) [e]	MPC (mg) [f]
ICM-103	160	4.0~6.0	60	130	>60	60
LA	315	2.5~4.0	0.1~1.0	< 5	< 8	10
LS	282	2.5~4.0	0.1	0.2	33	>50
DDNP	157	0.8~1.1	24.7	1.8	17	70

[a] Thermal stability; [b] Impact sensitivity; [c] Friction sensitivity; [d] Electrostatic discharge sensitivity; [e] Flame sensitivity; [f] Minimum primary charge. Data cited from ref. 20 and ref. 34. The normalized values in the paper is based the minimum sensitivity value.

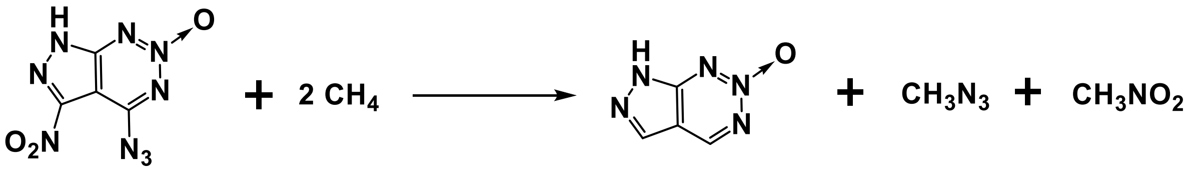

Supplementary Figure 17. Isodesmic reaction of ICM-103.

Supplementary Note 1. The theoretic calculation about enthalpy of formation of ICM-103

Theoretical calculations are performed by using the Gaussian 09 (Revision D.01) suite of programs [4] The geometric optimization and frequency analyses are completed by using the B3LYP functional with the 6-31+G** basis set. Single energy points are calculated at the MP2/6-311++G** level of theory. For all of the compounds, the optimized structures are characterized to be true local energy minima on the potential-energy surface without imaginary frequencies. The isodesmic reaction is carried out to obtain the gas-phase heat of formation of the neutral compound. The gas-phase enthalpies of the building-block molecules are obtained by using the atomization method with the G2 ab

initio calculations. Then the remaining task is to determine the solid-state heat of formation for the synthesized compound.

The solid-state enthalpy of formation for neutral compound can be estimated by subtracting the heat of sublimation from gas-phase heat of formation. On the basis of the literature [7], the heat of sublimation can be estimated with Trouton's rule according to supplementary equation 1, where T represents either the melting point or the decomposition temperature when no melting occurs prior to decomposition:

$$\Delta H_{\mathrm{sub}} = 188 / J\ mol^{-1} K^{-1} \times T \qquad (1)$$

In this work, the crystal calculation is done by using Crystal Explorer software [8]. The deformation potential can be expressed by energy induced pressure and the energy is obtained from the single point energy difference before and after deformation. For convenient comparison, the value is converted from mol units into volume units by dividing by the unit cell volume supplementary equation 2.

$$P = \left(E_{\mathrm{after\text{-}def}} - E_{before\text{-}def}\right) / V_{unit\text{-}cell} \qquad (2)$$

In this part of the calculations, single point energies are obtained from optimized structures using BLYP-D3/def2-QZVPP method using ORCA 3.0 [9,10].

Supplementary Note 2. Calculation about enthalpy of formation of ICM-103 by experiment

The constant-volume combustion energy of ICM-103 is determined by an oxygen bomb calorimetry (IKA C5000) [11]. Approximately 200 mg ICM-103 is pressed with a well-define amount of benzoic acid (ca. 800 mg) to form a tablet to ensure better combustion. The recorded data are the average of six single measurements. The calorimeter is calibrated by the combustion of certified benzoic acid in an oxygen atmosphere at a pressure of 30.5 bar. The experimental data of constant-volume combustion energy (six single measurements).

The averaged experimental value for the constant volume combustion energies ($\Delta_c U_m^\theta$) of ICM-103 is -11066 J g^{-1} (-2468.81 kJ mol^{-1}). The combustion reaction equation supplementary equation 3 and energy of combustion supplementary equation 4 are listed as follows.

The combustion reaction equation supplementary equation 3:

$$C_4HN_9O_3(\mathrm{s}) + \frac{11}{2}O_2(g) \rightarrow 4CO_2(g) + \frac{1}{2}H_2O(l) + \frac{9}{2}N_2 \qquad (3)$$

The energy of combustion equation supplementary equation 4:

$$\Delta_{\mathrm{c}} H_m^\theta (C_4HN_9O_3, s) = \Delta_{\mathrm{c}} U_m^\theta + \Delta nRT \qquad (4)$$

where Δn is the change in the number of gas products during the reaction process, *R* is 8.314×10^{-3} J mol^{-1} K^{-1}, and T is 298.15 K. According to supplementary equation 3 and supplementary equation 4, the calculated combustion enthalpy of ICM-103 is derived to be -2454.57 kJ mol^{-1}. Based on the calculated combustion enthalpy of ICM-103 and the known enthalpies of formation of the combustion products (CO_2 (g) = -393.51 kJ mol^{-1}), H_2O (l) = -285.83 kJ mol^{-1}, and N_2 is zero), the standard formation enthalpy of ICM-103 is back-calculated from its combustion equation

supplementary equation 3. On the basis of Hess's law in thermochemical supplementary equation 5, the standard enthalpy of formation ($\Delta_f H_m^\theta$) of ICM-103 is calculated to be 739.40 kJ mol^{-1}.

$$\Delta_f H_m^\theta(C_4HN_9O_3,s) = 4\Delta_f H_m^\theta(CO_2,g) + \frac{1}{2}\Delta_f H_m^\theta(H_2O,l) + \Delta_c H_m^\theta(C_2H_2N_8O_6,s) \quad (5)$$

Supplementary Table 22 Calculated detonation parameters for ICM-103.

Items	D [km·s^{-1}]	P [GPa]	Q [kJ·kg^{-1}]	T [K]	V [L·kg^{-1}]	OB [%]	ρ[g·cm^{-3}]	$\Delta_f H_m^\theta$[kJ ·mol^{-1}]
Values	9.11	35.14	-5964.86	4347	709	-39.44	1.86	744.75

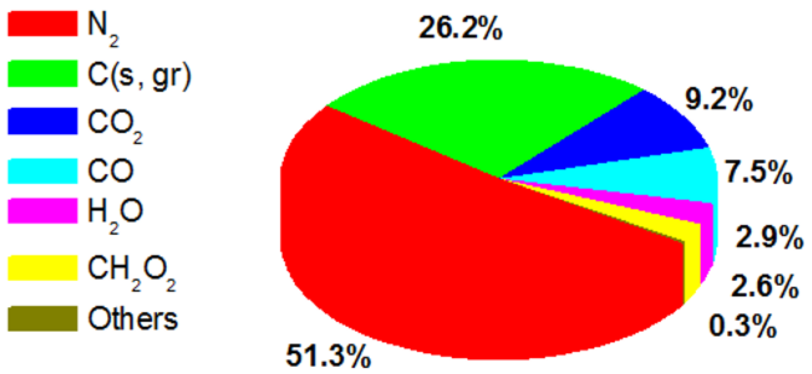

Product	mol%
N_2	51.3
C(s,gr)	26.2
CO_2	9.2
CO	7.5
H_2O	2.9
CH_2O_2	2.6
Others	0.3

Supplementary Figure 18. Detonation products of ICM-103.

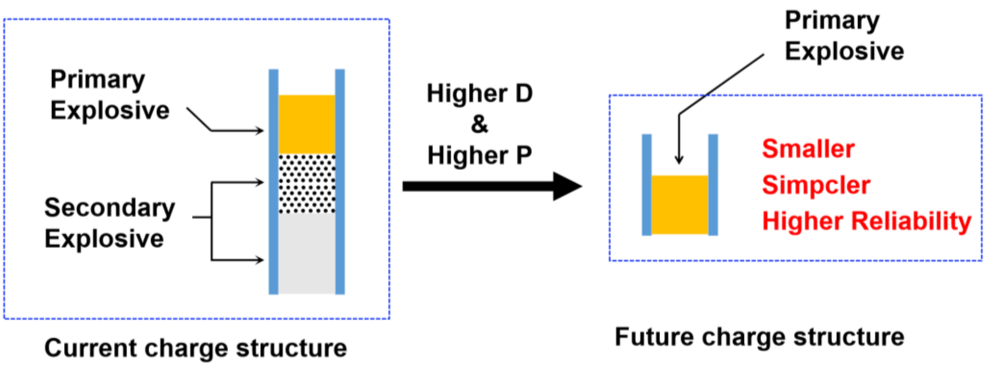

Supplementary Figure 19. The possible change of charge structure resulted by high detonation properties. it should be pointed out that high D and P values of ICM-103 seemed very favorable for simplifying the charge structure, thereby improving the reliability of initiating explosive devices (IEDs). As shown, the charge structure of current IEDs is the primary explosive, loose secondary explosive (RDX or PETN) and compacted secondary explosive (RDX or PETN) from top to bottom. If the detonation performances of primary explosives reaches the level of secondary explosives, people will have the opportunity to design single-layer charge to replace the currently used multi-layer charge.

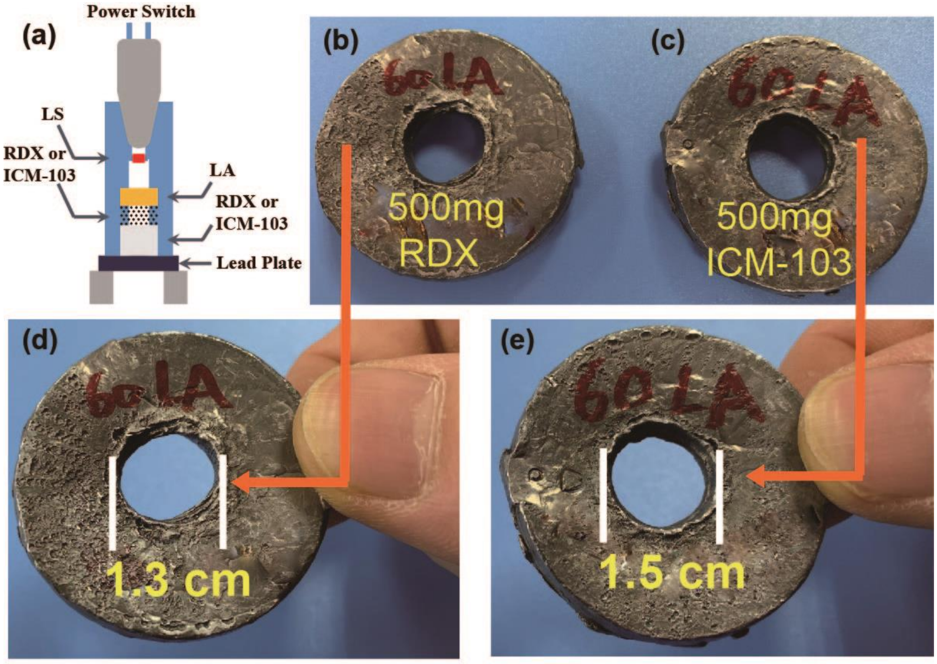

Supplementary Figure 20. The detonation test of ICM-103. 60 mg LA is used as primary explosive to detonate 500 mg RDX or 500 mg ICM-103 with a pyrotechnical igniter. As shown in b) and c), both RDX and ICM-103 as secondary explosives can be initiated successfully using the LA as primary explosive. The aperture of perforated lead plate in the test of using RDX and ICM-103 separately is ca. 1.3 cm and ca. 1.5 cm, respectively, demonstrating that ICM-103 shows the higher detonation performances than that of RDX. a) Diagram of the test setup with 60 mg LA and 500 mg RDX or ICM-103 samples with pyrotechnical igniter to ignite; b)-e) Perforated lead plate as the result of detonation tests: b) and d) 60 mg LA and 500 mg RDX; c) and e) 60 mg LA and 500 mg ICM-103.

Supplementary References

1. Huynh, M. H. V., Hiskey, M. A., Chavez D. E., Naud D. L. & Gilardi R. D. Synthesis, Characterization, and Energetic Properties of Diazido Heteroaromatic High-Nitrogen C−N Compound. *J. Am. Chem. Soc.* **127**, 12537-12543(2005).
2. Hao, W., Zhang, J. & Shreeve J. M. Synthesis, characterization, and energetic properties of 6-aminotetrazolo[1,5-b]-1,2,4,5-tetrazine-7-N- oxide: a nitrogen-rich material with high density. *Chem. Asian J.* **10**, 1130-1132(2015).
3. Chavez, D. E., Parrish, D. A., Mitchell, L. & Imler, G. H. Azido and tetrazolo 1,2,4,5-tetrazine N-oxides. *Angew. Chem. Int. Ed.* **56**, 3575-3578(2017).
4. Frisch, M. J. et al., *Gaussian 09, Revision D. 01*, Gaussian Inc., Wallingford C T (2009).
5. Miehlich, B., Savin, A., Stoll, H. & Preuss, H. Results obtained with the correlation energy density functionals of becke and Lee, Yang and ParrChem. *Phys. Lett.* **157**, 200(1989).
6. Lee, C. T., Yang, W. T. & Parr, R. G. Development of the colle-salvetti correlation-energy formula into a functional of the electron density. *Phys. Rev. B* **37**, 785(1988).

7. Westwell, M. S., Searle, M. S., Wales, D. J., Wiliams, D. H. Empirical Correlations between ThermodynamicProperties and Intermolecular Forces. *J. Am. Chem. Soc.* **117**, 5013-5015 (1995).
8. Wolff, S. K., Grimwood, D. J., McKinnon, J. J., Turner, M. J., Jayatilaka, D., Spackman, M. A. Crystal Explorer,version 3.1; *University of Western Australia: Crawley*, Australia (2012).
9. Grimme, S., Ehrlich, S., Goerigk, L. Effect of the damping function in dispersion corrected density functional theory. *J. Comput. Chem.* **32**, 1456-1465 (2011).
10. Neese, F. The ORCA program system. *WIREs Comput. Mol. Sci.* **2**, 73-78 (2012).
11. Feng, Y., Bi, Y., Zhao, W., Zhang, T. Anionic Metal-Organic Frameworks Lead the Way to Eco-Friendly High-Energy-Density Materials. *J. Mater. Chem. A* **4**, 7596-7600 (2016).
